# Synthesis of [^3^H]muscimol

**DOI:** 10.1002/jlcr.4159

**Published:** 2025-08-11

**Authors:** Michal Kriegelstein, Aleš Marek

**Affiliations:** ^1^ Institute of Organic Chemistry and Biochemistry Czech Academy of Sciences Prague Czechia

**Keywords:** [^3^H]muscimol, ^3^H NMR, B^3^H_3_, GABA, tritioborane, tritium

## Abstract

Muscimol, a potent GABA_A_ receptor agonist and psychoactive alkaloid found in *Amanita* mushrooms, is widely used as a tool compound in neurochemical research. Despite its importance, synthetic access to [^3^H]muscimol of high specific activity has remained limited due to the challenges associated with conventional labeling strategies. Herein, we report a novel synthetic approach for the preparation of [^3^H]muscimol based on the reduction of a suitably protected amide precursor using in situ generated tritioborane (BT_3_·THF). The precursor was synthesized in four steps from dimethyl acetylenedicarboxylate, and subsequent electrophilic reduction afforded [^3^H]benzyl‐protected muscimol in a radiochemical yield of 44 mCi (1.63 GBq) and a molar activity of 48.3 Ci/mmol (1.79 TBq/mmol). Final deprotection with HBr in acetic acid yielded [^3^H]muscimol·HBr in > 95% radiochemical purity. The method avoids the use of bulk tritiated water employed in established synthetic protocols and enables safe, reliable, and efficient access to this valuable radioligand for applications in GABA receptor studies.

## Graphical abstract

A novel synthetic approach for the preparation of [^3^H]muscimol (48.3 Ci/mmol, 1.8 TBq/mmol) based on the reduction of a suitably protected amide precursor using in situ generated tritioborane (BT_3_·THF) is reported. The method avoids the use of bulk tritiated water employed in established synthetic protocols and enables safe, reliable, and efficient access to this valuable radioligand for applications in GABA receptor studies.

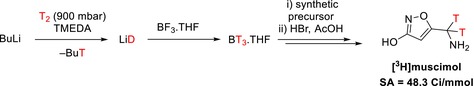

## 1 INTRODUCTION

**FIGURE 1:**
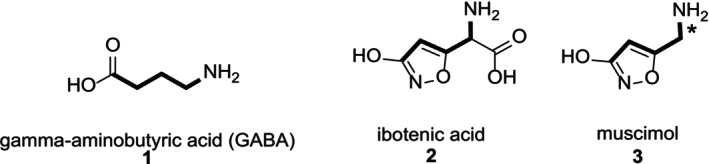
GABA receptor binding ligands.

Muscimol (**3**, also known as agarin or pantherine, 5‐aminomethyl‐3‐hydroxyisoxazole) is a structural analog of γ‐aminobutyric acid (**1**, GABA). It is the principal psychoactive alkaloid present in various mushrooms of the *Amanita* genus and exhibits high‐affinity binding to the GABA site of GABA_A_ receptors [1, 2] Naturally co‐occurring in many fungal species with muscarine, muscazone, and the psychoactive isoxazole ibotenic acid (**2**, (*S*)‐2‐amino‐2‐(3‐hydroxyisoxazol‐5‐yl)acetic acid), muscimol arises as a product of ibotenic acid's spontaneous decarboxylation. Because of its potent activity as a central nervous system (CNS) depressant and GABA agonist, muscimol has been widely employed as a tool for studying GABA receptors [3]. Unlike other GABAergic agents such as barbiturates or benzodiazepines, muscimol binds directly to the same site as GABA itself [4]. It has been recently identified as an inhibitor of membrane rupture during pyroptosis [5], and its derivatives have been investigated as possible insecticides [6]. Furthermore, compound **3** has shown promise in the treatment of neuropathic pain [7]. Although not clinically approved, tritiated **3** has been extensively utilized as a valuable tool in neuroscience, particularly in GABA receptor research [8, 9, 10, 12]. Remarkably, tritiated **3** has been employed in photoaffinity labeling studies, in which it becomes irreversibly incorporated into the adjacent receptor protein [13, 14]. The structure of **3** has also inspired the design of GABA uptake inhibitors and GABA_A_ receptor agonists [15]. Based on these findings, the development of a straightforward synthesis of isotopically labeled **3** remains an important goal (Figure 1) [16].

The synthesis of [^3^H]‐**3** was already reported by Filer et al. [17] via the spontaneous decarboxylation of ibotenic acid (**2**) in the presence of ^3^H_2_O in DMSO. However, this method requires access to a large stock solution of ^3^H_2_O—about 100 Ci (3.7 TBq) with molar activity (M.A.) of 58.0 Ci/mmol (2.15 TBq/mmol). The desired radioligand [^3^H_1_]‐**3** was reported to be isolated with M.A. of 29.5 mCi/mmol (1.09 GBq/mmol) and a total activity of 109 mCi (4.03 GBq), corresponding to a radiochemical yield (RCY) of 0.1% [17]. In a later publication, [^3^H]‐**3** was prepared using the same approach, affording a higher M.A. of 37.8 mCi/mmol (1.40 GBq/mmol) [18]. Although the isolated quantity and achieved M.A. are undoubtedly satisfactory [17], conducting a single labeling experiment with such an extraordinary amount of absolute ^3^H_2_O is inconsistent with modern trends in radiochemical synthesis, which emphasize minimizing radioactive material for reasons of safety, toxicity, cost, and waste production.

Moreover, in light of the very low isolated RCY (0.1%) [17] of [^3^H]‐**3** reported for this established method (calculated on the amount of ^3^H_2_O used), we considered alternative and more reliable labeling strategies. Given the challenging and low‐yield generation of highly toxic and unstable super‐heavy water (^3^H_2_O), we pursued a synthetic approach based on the reduction of a suitable precursor with a high‐quality tritiated reagent. Borotritides can be routinely synthesized in situ from carrier‐free tritium gas, thus avoiding the need to handle ^3^H_2_O generation. Although ^3^H_2_O can also be generated in situ from PdO or PtO [19], the residual Pd/Pt metals may interfere with subsequent reactions. This necessitates careful manipulation to remove hydrogen‐activated Pd[0]/Pt[0] species, which could act as undesired reducing agents, especially in the presence of sensitive functional groups on the target molecule. The trace amounts of the generated ^3^H_2_O (as droplets) are typically either transferred to a separate vial via microdistillation or diluted in dry co‐solvent (e.g., 1,4‐dioxane), with the residual solid Pd[0]/Pt[0] catalyst removed by a syringe filter.

The analysis and preparative purification of very small and highly polar organic compounds has long posed a challenge. Tritiated **3** (C_4_H_6_N_2_O_2_) was reported to be purified by preparative thin‐layer chromatography (TLC) using BuOH/H_2_O/HOAc eluent system [17]. However, the subsequent workup of the eluted TLC—scraping the silica gel to recover the product—was considered unsafe because of the risk of radioactive dust generation and potential workplace contamination. As an alternative, hydrophilic interaction liquid chromatography (HILIC) represents the method of choice for the purification of highly polar analytes. Unfortunately, compound **3** exhibits poor solubility in the high‐organic/low‐water solvent mixtures typically employed in HILIC separations. In general, reduction of amide functional group can introduce up to two heavy hydrogen atoms per molecule, leading to high M.A. of the product—theoretically up to 58 Ci/mmol (2.15 GBq/mmol). Furthermore, the use of a suitably protected precursor could enhance the lipophilicity of the labeled compound, thus facilitating purification by conventional reverse‐phase liquid chromatography. Several one‐step syntheses of appropriate precursor, followed by reduction to desired **3**, have already been reported [20, 21, 22].

Several methods for the generation of B^3^H_3_ were reported in the literature, beginning with NaB^3^H_4_ [23, 24], which is commercially available (e.g., from RC Tritec). The synthesis of NaB^3^H_4_ has been achieved via direct H–T exchange on borohydrides [25, 26], although heating reaction vessels containing radioactive hydrogen gas to temperatures exceeding 300°C presents significant safety concerns. Than et al. described the direct formation of B^3^H_3_ by the reaction of BF_3_ with in situ generated Li^3^H, prepared from tritium gas, *n*‐BuLi, and *N*,*N*,*N′*,*N′*‐tetramethylethylenediamine (TMEDA) [27]. In contrast to the synthesis of other boron species such as LiB^3^H(OMe)_3_ [28], this approach required the removal of volatile reagents (TMEDA, *n*‐BuT) prior to the reaction of Li^3^H with BF_3_.

## 2 Results

**SCHEME 1:**
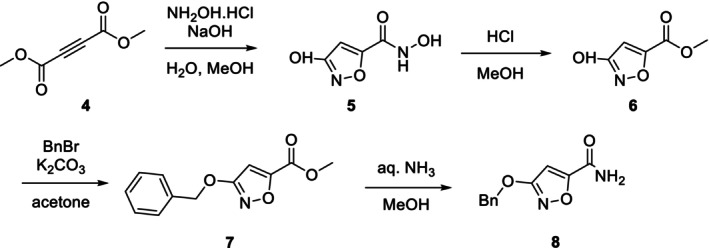
Synthetic pathway to the key intermediate **8**.

The key intermediate was prepared following the modified literature procedures (Scheme 1) [6, 20]. Dimethyl acetylenedicarboxylate (**4**) was condensed with hydroxylamine to afford isoxazole derivative **5** in 76% yield after multiple precipitation steps. The resulting hydroxamic acid was hydrolyzed with methanolic HCl under reflux to give methyl ester **6** in 54% yield. Compound **6** was then protected with benzyl bromide in acetone at elevated temperature, and the resulting product **7** was isolated in 50% yield after column chromatography. This protected ester was converted into amide **8** via straightforward ammonolysis with aqueous ammonia in methanol in 76% yield (corresponding to a 17% overall yield over four steps).

Once the key intermediate **8** was in our hands, the reduction of its amide group to the corresponding α‐tritiated amine appeared simple using electrophilic tritiated borane (BF_3_·THF) [29]. To evaluate this approach, the formation of freshly generated deuteroborane (B^2^H_3_·THF) with high deuterium enrichment was first investigated [6]. Initially, we attempted to adapt a straightforward method for generating deuteroborane, avoiding the lyophilization of volatile compounds (e.g., TMEDA, *n*‐hexanes, and *n*‐Bu^2^H) after the formation of Li^2^H—an approach commonly used for the preparation of nucleophilic boranes) [28]. Lewis acid BF_3_·THF was added directly to the crude Li^2^H reaction mixture, and the reduction of compound **8** was attempted at room temperature. However, under these conditions, the formation of B^2^H_3_·THF could not be confirmed, as no reduction of the amide bond was observed upon addition of a THF solution of **8** (Table 1, entry 1). Ultimately, the lyophilization of volatile components—especially basic TMEDA—from the reaction mixture prior to the addition of acidic BF_3_·THF reagent proved to be essential.

**TABLE 1:** Optimization of BD_3_ reduction of intermediate **8**.

Entry	Hydrogen pressure (mbar)	BuLi/TMEDA/BF_3_ [Equiv]	*T* (°C)	*t* (h)	Result
1	650	10:11:13.5	RT	18	No reaction (no lyophilization of volatiles prior to BF_3_ addition)
2	650	10:11:13.5	RT → reflux	18	55% conversion to **9**
3	650	30:33:40.5	Reflux	18	Full conversion to **9**
4	900	30:33:40.5	Reflux	18	Tritiation experiment, 20% conversion to **9**

**SCHEME 2:**
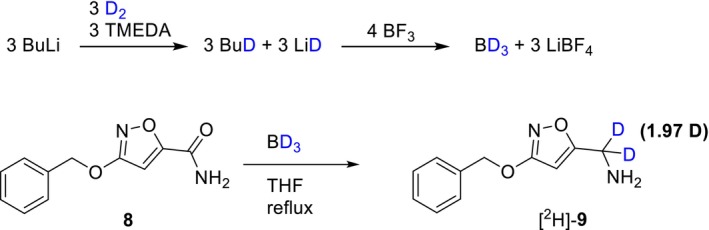
Generation of B^2^H_3_ and reduction of the amide **8**.

When the procedure was repeated with thorough lyophilization of all volatiles prior to the addition of BF_3_ [27], only traces of product **9** were detected by LC‐MS analysis of the reaction mixture. To further improve the conversion of **8**, the reaction temperature was increased to the reflux of THF, resulting in significant—but still incomplete—conversion of **8** (55%). Taking into account the generally low yield of Li^2^H formation (approx. 30%–40%) [28, 30, 31] and the stoichiometry of the B^2^H_3_ generation (Scheme 2, top), it was calculated that only 1.5 equivalents of B^2^H_3_ could realistically be formed under these conditions (Table 1, entry 2). To ensure full conversion of **8**, the equivalents of all reagents involved in the reaction (Scheme 2, bottom) were increased threefold (Table 1, entry 3). The degree of deuterium incorporation in [^2^H]‐**9** was determined by ESI^+^‐MS to be 1.97 D‐atoms per molecule, which theoretically corresponds to a M.A. of 57 Ci/mmol (2.1 TBq/mmol) for the tritiated analog, assuming use of tritium gas.

**SCHEME 3:**
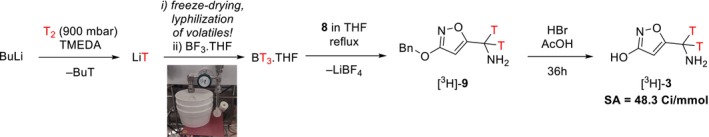
[^3^H]muscimol synthesis via in situ formation of BT_3_·THF and reduction of amide **8**.

With suitable conditions established for both the generation of deuterated borane and the reduction of **8** (Scheme 2), tritiated borane (B^3^H_3_·THF) was generated using an analogous procedure with carrier‐free tritium gas (Table 1, entry 4) (Scheme 3). Nevertheless, even with an excess of freshly generated B^3^H_3_, only 20% conversion of **8** was observed after 24 h. We assume this was caused by the kinetic isotope effect. Either the formation of B^3^H_3_·THF did not fully form as expected, and/or the amide reduction was proceeding slower with B^3^H_3_·THF compared with B^2^H_3_·THF. Possibly, a longer reaction time could increase the conversion. As we have isolated a substantial amount of [^3^H]‐**9**, we decided not to further optimize the reaction using tritium gas due to time and costs associated. Despite this limited conversion, the outcome was considered sufficient to proceed to the final step (Scheme 3).

**FIGURE 2:**
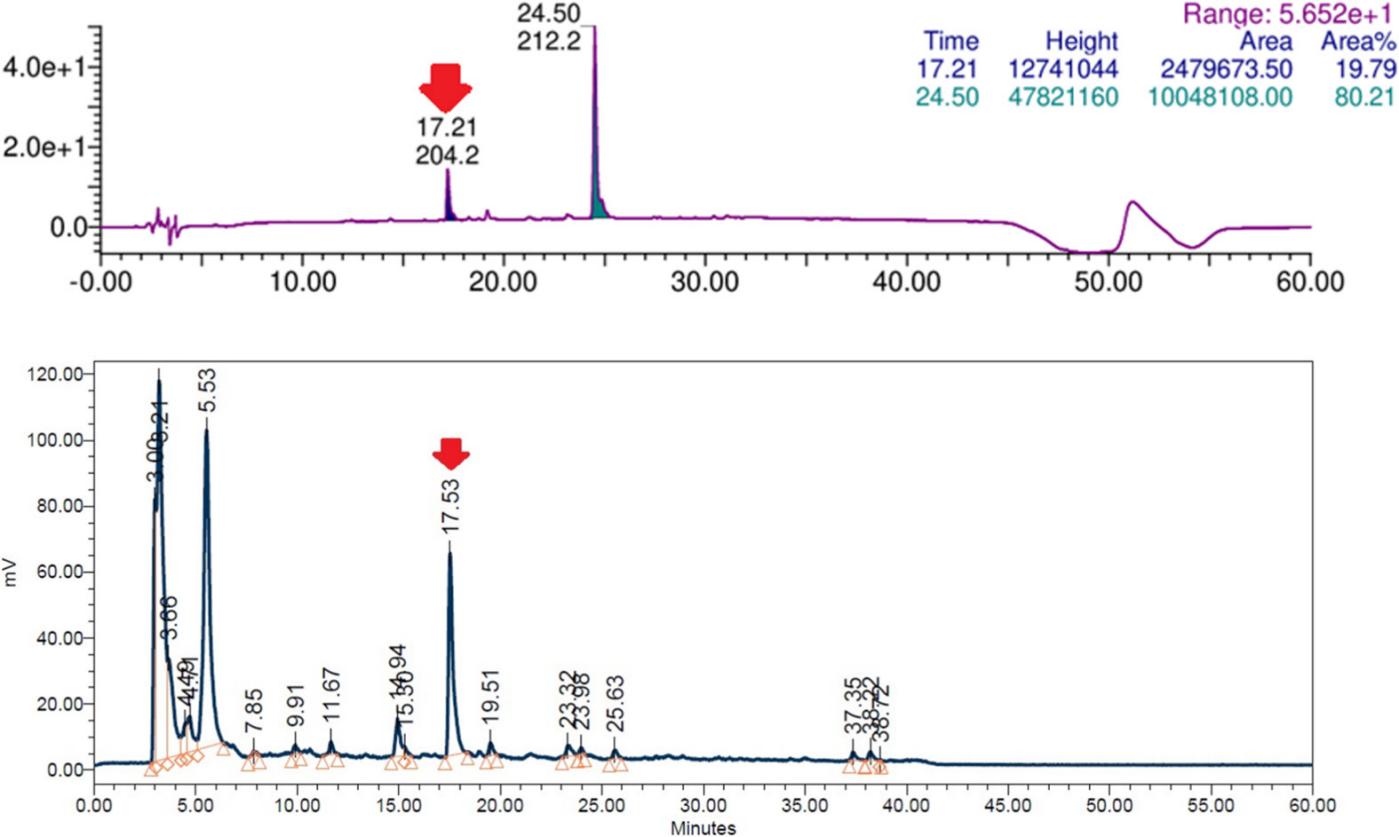
HPLC‐UV chromatogram (255 nm) of crude reaction mixture of [^3^H]‐**9** (top); radio‐HPLC chromatogram of the reaction mixture (bottom), the arrows at 17.2 and 17.5 min show peaks of [^3^H]‐**9**.

Following quenching of unreacted reagent by the addition of aqueous HCl and subsequent lyophilization to remove labile activity, a RCY of 220 mCi (8.14 GBq, 11%) for crude benzylated muscimol [^3^H]‐**9** was determined by radio‐HPLC. This crude product was dissolved in MeOH (10 mL) and purified in multiple small‐scale batches, yielding 44 mCi (1.68 GBq) of pure [^3^H]‐**9**. An aliquot (9.7 mCi, 359 MBq) of this intermediate was fully deprotected by treatment with HBr in acetic acid, yielding 6.4 mCi (237 MBq) of muscimol hydrobromide [^3^H]‐**3**·HBr after 36 h at room temperature. The M.A. of [^3^H]‐**3** was determined by ESI^+^‐MS to be 48.3 Ci/mmol (1.79 TBq/mmol, 1.66 T‐atoms per molecule). A structural characterization of the labeled product was performed using radio‐HPLC, radio‐TLC, and ^1^H/^3^H NMR (Figure 2).

**FIGURE 3:**
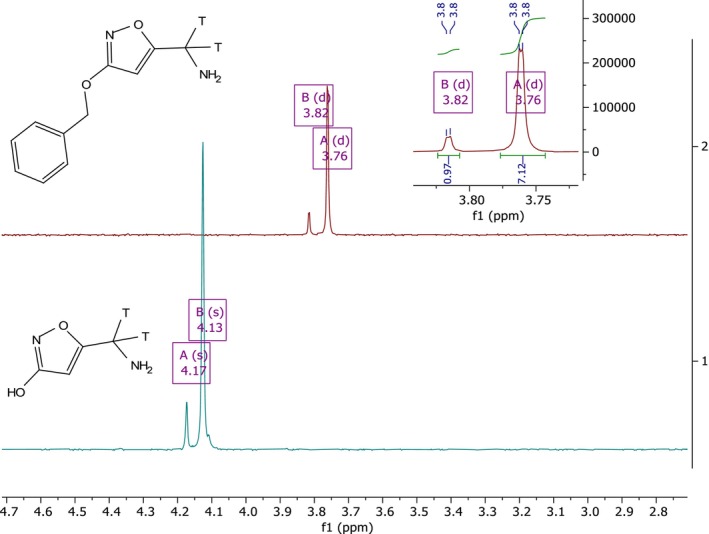
^3^H‐NMR spectrum of tritiated precursor [^3^H]‐**9** in CD_4_OD (top) and of [^3^H]muscimol in D_2_O (bottom).

Interestingly, two distinct peaks were observed in ^3^H NMR spectra of both the precursor [^3^H]‐**9** and the final product [^3^H]‐**3** (Figure 3). These signals are attributed to the presence of singly and doubly tritiated species. A similar behavior was recently observed during the synthesis of variously deuterated methyl groups of pirtobrutinib‐*d*_7_ [32]. Indeed, the coexistence of mono‐ and poly‐labeled functional groups has been documented in the literature [33], including in tritiated muscimol prepared via decarboxylation of ibotenic acid (**1**) [18]. The doublets observed in the NMR spectra of the protected precursor **9** (Figure 3, top) are attributed to a four‐bond coupling between C‐4 and C‐7 ([4] *J*_HT_ = 0.9 Hz) as previously reported by Kupka [34].

## 3 Conclusion

In conclusion, we have developed a novel synthetic approach for the preparation of tritiated muscimol with high molar activity, based on the reduction of an amide precursor using in situ generated tritioborane. The required precursor was prepared in four consecutive steps with an overall yield of 17%. Reduction of the protected amide intermediate with freshly generated B^3^H_3_·THF afforded tritiated benzyl muscimol in a satisfactory RCY (44 mCi, 1.63 GBq) and molar activity (48.3 Ci/mmol, 1.8 TBq/mmol). The final deprotection with HBr furnished pure [^3^H]muscimol·HBr with the same molar activity and in high radiochemical purity (> 95%).

## 4 Experimental

### 4.1 General

Unless otherwise specified, all reactions were conducted under ambient conditions without special precautions to exclude moisture or air. Reaction work‐ups and column chromatography were carried out using commercially available solvents (Acros) without further purification. Dry THF was freshly distilled from sodium/benzophenone prior to use.

^1^H and ^13^C NMR spectra were recorded on either a Bruker Avance III (400 and 101 MHz) spectrometer equipped with a nitrogen‐cooled broadband 5‐mm probe or a Bruker Avance II (300 and 75 MHz) instrument, using CDCl_3_, DMSO‐*d*_6_, MeOH‐*d*_4_, or D_2_O as solvents. Spectra were referenced to the residual nondeuterated solvent peaks: δ = 7.26 and 77.16 ppm for CDCl_3_, 2.50 and 39.52 ppm for DMSO‐*d*_6_, and 3.31 ppm for MeOH‐*d*_4_ (^1^H only). ^3^H NMR spectra were acquired without referencing on a Bruker Avance II (320 MHz) instrument in MeOH‐*d*_4_ or D_2_O. All ^13^C NMR spectra were acquired with broadband ^1^H decoupling. NMR data are reported as follows: chemical shift *δ* (in ppm), coupling constants *J* (in Hz), and peak integration. Multiplicities are indicated using the following abbreviations: s (singlet), d (doublet), t (triplet), q (quartet), m (multiplet), app (appears as), and br (broad).

Analytical TLC was performed on Kieselgel 60 F_254_ plates (Merck). Compounds were visualized under UV light (254 and 365 nm) and by staining with basic KMnO_4_ solution followed by heating. Column chromatography was conducted on SiO_2_ 60 (particle size 0.040–0.063 mm, 230–400 mesh; Merck) using commercially available solvents and air pressure to accelerate flow.

The high‐resolution mass spectroscopy (HRMS) in the ESI^+^ mode was performed on an LTQ Orbitrap XLc (Thermo Fisher Scientific). HRMS in the EI^+^ mode was performed on a 7250 GC/Q‐TOF system (Agilent).

The HPLC analyses were performed on an Alliance e2695 (Waters) instrument combined either with a single quadrupole mass detector SQ Detector 2 (Waters) for HPLC‐MS using ESI^+^ ionization mode, or with a radioactivity flow detector Ramona Star (Elysia‐Raytest) for radio‐HPLC. A liquid scintillator was added prior to radio‐detection.

Deuteration reactions were performed on a deuteration manifold (RC Tritec AG) using deuterium gas (99.8% D‐atom, Sigma‐Aldrich). Tritiation reactions were performed on a tritiation manifold system (RC Tritec AG) using UT_3_‐bed technology for carrier‐free tritium gas (purity min 99%, specific activity 2.58 Ci/mL). Liquid scintillation counting (LSC) was performed using Tri‐Carb 2900TR (PerkinElmer). Chemical reagents were purchased from commercial suppliers: dimethyl acetylenedicarboxylate, deuterium gas, benzyl bromide, BF_3_·THF, BH_3_·THF, *n*‐BuLi, TMEDA (Sigma‐Aldrich); aqueous HBr (Penta); and ammonium hydroxide (Lach‐Ner).

***N*,3‐Dihydroxy‐1,2‐oxazole‐5‐carboxamide (5):** NaOH (2 g, 50 mmol, 5 eq.) was dissolved in water (10.0 mL) and NH_2_OH.HCl (1740 mg, 25 mmol, 2.5 eq.) was added. This solution was cooled in an ice bath, and DMAD (1.2 mL, 10 mmol, 1 eq.) in MeOH (3 mL) was added dropwise with rapid stirring. The reaction mixture turned dark orange/red/brown and was stirred at room temperature for 24 h. The next day, the reaction mixture was washed with DCM (3 × 10 mL). The aqueous solution was acidified with 5% aq. HCl to pH 2 and evaporated to dryness. The dark orange residue was suspended in EtOH (30 mL), and solids were removed by filtration. The filtrate was evaporated to provide dark residue (1.8 g). This residue was suspended in cold ACN (10 mL). Solids were removed by filtration and dried under vacuum to provide 1.1 g of brown solid (76%).

^1^H NMR (401 MHz, DMSO‐D_6_) δ 11.65 (s, 2H), 9.49 (br. s, 1H), 6.56 (br. s, 1H).

^13^C NMR{^1^H} (101 MHz, DMSO‐D_6_) δ 170.4, 162.1, 153.9, 97.4.

HRMS (ESI−) calc. for C_4_H_3_O_4_N_2_: 143.00983, found: 143.00965.

**Methyl 3‐hydroxyisoxazole‐5‐carboxylate (6):** *N*,3‐dihydroxy‐1,2‐oxazole‐5‐carboxamide (**5**) (879.0 mg, 6.1 mmol, 1 eq.) was dissolved in 1.8 M methanolic HCl (85 mL, 152.5 mmol, 25 eq.) and refluxed until TLC (BuOH/HOAc/H_2_O 25:4:10) revealed complete conversion (visualization by UV and 1% FeCl_3_). The reaction mixture was then evaporated under reduced pressure. The residue was suspended in diethyl ether (50 mL) and washed three times with water (1 × 50, 2 × 30 mL). Combined water layers were extracted with diethyl ether (20 mL). Combined ether extracts were washed with brine, dried with MgSO_4_, and evaporated to yield 473 mg of brown solid (54%).

^1^H NMR (401 MHz, DMSO‐D_6_) δ 6.75 (s, 1H), 3.86 (s, 3H).

^13^C NMR{^1^H} (101 MHz, DMSO‐D_6_) δ 170.6, 159.4, 156.8, 101.2, 52.8.

HRMS (ESI−) calc. for C_5_H_4_O_4_N: 142.01458, found: 142.01431.

**Methyl 3‐(benzyloxy)isoxazole‐5‐carboxylate (7):** A thick‐walled microwave vial (25 mL) was charged with K_2_CO_3_ (1015 mg, 7.3 mmol, 2 eq.), and a solution of methyl 3‐hydroxyisoxazole‐5‐carboxylate (**6**) (533 mg, 3.7 mmol, 1 eq.) in acetone (10.0 mL) was added. The vial was sealed and heated in a DrySyn block at 70°C for 60 min. Then, the temperature was lowered to 50°C, and benzyl bromide (0.65 mL, 5.5 mmol, 1.5 eq.) in acetone was added (backpressure), and the reaction was kept at 50°C overnight. Formed solids were filtered through celite, and the celite was washed with acetone (3×). The filtrate was evaporated and suspended in water (50 mL). The water mixture was extracted with EtOAc (3 × 50 mL). Combined organic extracts were washed with brine, dried with MgSO_4_, and evaporated. The residue was purified by flash chromatography (100% Cyclohexane → 90% cyclohexane:10% EtOAc) to provide 479 mg (55%) of white solid after drying in vacuum.

^1^H NMR (401 MHz, CDCl_3_) δ 7.47–7.34 (m, 5H), 6.57 (s, 1H), 5.32 (s, 2H), 3.95 (s, 3H).

^13^C NMR{^1^H} (101 MHz, CDCl_3_) δ 171.4, 160.5, 157.2, 135.3, 128.9, 128.8, 128.5, 101.0, 72.3, 53.0.

HRMS (ESI−) calc. for C_12_H_11_O_4_NNa: 256.05803, found: 256.05827.

**3‐(benzyloxy)isoxazole‐5‐carboxamide (8):** Methyl‐3‐(benzyloxy)isoxazole‐5‐carboxylate (479 mg, 2.0 mmol) was dissolved in MeOH (3.0 mL), and aq. NH_3_ (27 mL) was added. A white precipitate was formed immediately; the suspension was kept stirred at room temperature overnight. The solvent was evaporated under reduced pressure, and water (20 mL) was added to the whitish residue. The precipitate was filtered and dried under reduced pressure, affording 335 mg of the product **8** (75%) as a white solid.

^1^H NMR (401 MHz, CDCl_3_) δ 7.48–7.34 (m, 5H), 6.59 (s, 1H), 6.42 (br. s, 1H), 5.90 (br. s, 1H), 5.31 (s, 2H).

^13^C NMR{^1^H} (101 MHz, CDCl_3_) δ 172.0, 163.2, 157.3, 135.3, 128.9, 128.8, 128.4, 99.5, 72.3.

HRMS (ESI+) calc. for C_11_H_10_O_3_N_2_Na: 241.05836, found: 241.05830.

**(3‐(Benzyloxy)isoxazol‐5‐yl)‐D**_**2**_ **‐methanamine ([** ^**2**^ **H]‐9):** An oven‐dried (130°C, 2 h) two‐necked deuteration flask with a stir bar was cooled under a flow of nitrogen and subsequently connected to a deuteration manifold. To the second arm of the manifold, a one‐necked flask was connected as well. The whole apparatus was evacuated for 15 min, flushed with nitrogen, and evacuated, and the connection between flasks was closed.

Deuterium gas was introduced (650 mbar), and then 1.6 M BuLi in hexanes (430 μL, 0.687 mmol, 30 eq.) was added via syringe. To the rapidly stirred solution of BuLi, TMEDA (113 μL, 0.756 mmol, 33 eq.) was added dropwise. A white precipitate immediately formed after the first drop of TMEDA and then dissolved after further addition of TMEDA, forming a yellowish cloudy solution. This solution was stirred at room temperature for 60 min, during which a white precipitate formed. Then, the reaction mixture was frozen in LN_2_, and the whole system was evacuated, backfilled with nitrogen, and evacuated again. The liquids were lyophilized into the second flask connected to the manifold. The residue was kept under vacuum for 45 min. The reaction flask was then filled with nitrogen, the residue was dissolved in dry THF (0.5 mL), and BF_3_·THF (102 μL, 928 μmol, 40.5 eq.) was added to the reaction mixture, and the reaction was heated in an oil bath at 70°C for 60 min. The reaction was cooled in an ice bath, and a solution of 3‐(benzyloxy)isoxazole‐5‐carboxamide (**8**) (5.0 mg, 23 μmol, 1 eq.) in freshly distilled THF (0.5 mL) was added dropwise, and the reaction was warmed up and stirred at 70°C overnight. The reaction was cooled to room temperature, quenched with 0.5 mL of 5% HCl, and evaporated.

LRMS (ESI+): 206.1 (4.21%), 207.1 (100%), 208.1 (13.34%).

**(3‐(Benzyloxy)isoxazol‐5‐yl)‐T**_**2**_ **‐methanamine ([** ^**3**^ **H]‐9):** An oven‐dried (130°C, 100 min) two‐necked deuteration flask with stir bar was cooled under a flow of nitrogen and then connected to the tritiation manifold (RC Tritec). The whole manifold was evacuated and kept under vacuum for 15 min. Tritium gas was released (1000 mbar) and introduced into the reaction flask. *n*‐BuLi (1.6 M) in hexanes (0.43 mL, 687 μmol, 30 eq.) was then added and vigorously stirred. Subsequently, TMEDA (115 μL, 756 μmol, 33 eq.) was added at once, forming a yellowish cloudy solution. After 60 min, the reaction was frozen in liquid nitrogen, and a residual tritium gas was re‐adsorbed onto the uranium bed. The volatiles (^3^H‐BuH, TMEDA, solvents) were lyophilized away into a second flask, and the remaining LiT was dried under high vacuum for 60 min. The reaction apparatus was filled with N_2_, and the white‐grey residue of LiT was dissolved in freshly distilled THF (0.5 mL), followed by the addition of BF_3_·THF (0.1 mL, 0.93 mmol, 40.5 eq.). The solution was then heated at 70°C, forming a grey cloudy mixture. After 60 min of heating, the reaction was cooled to room temperature, and a solution of the amide (5.0 mg, 23 μmol, 1 eq.) in freshly distilled THF (0.8 mL) was added, and the reaction was heated at 70°C for 16 h.

After 16 h, the reaction was cooled to room temperature, forming a cloudy suspension that was quenched with a 5% aqueous HCl solution (0.5 mL), evolving gas and foaming. This mixture was stirred at room temperature for 60 min. The reaction solution was transferred into a lyophilization flask, and the reaction vessel was washed with MeOH (5 × 1 mL). The combined solution was lyophilized to remove labile activity. The white residue after lyophilization was dissolved (not all material dissolved, white solids, likely inorganic salts were undissolved) in MeOH (5 mL). An aliquot was taken for analysis (2085 mCi of crude mixture was isolated, ca 20% conversion, Figure 2), and the remaining solution was lyophilized twice from MeOH. The remaining residue was stored as a suspension in MeOH (10 mL) at −20°C. The crude product [^3^H]‐**9** was purified by liquid chromatography (**METHOD 1**), yielding 44 mCi of [^3^H]‐**9**. This precursor was stored at 1.2 mCi/mL in EtOH in a cryostation (−196°C).

9.7 mCi of the intermediate **9** were dissolved in acetic acid (250 μL), and 50% HBr was added (250 μL). The solution was gently shaken at room temperature for 36 h until radio‐LC revealed full conversion of **9**. Solvents were removed under reduced pressure, and the residue was lyophilized from water (3 × 1 mL) and EtOH (2 × 1 mL). After the last lyophilization, the residue was dissolved in water (0.5 mL), extracted with hexane (3 × 0.5 mL), and the aqueous layer was lyophilized to obtain 6.4 mCi of the product [^3^H]‐**3**. This product was formulated at 1 mCi/mL in H_2_O/EtOH. Samples were stored in liquid nitrogen (−196°C), whereas small aliquots were stored at −20°C and −80°C for stability testing (**METHOD 2**). No significant decomposition was observed at −80°C and −196°C after 16 months.

**[** ^**3**^ **H]Bn‐muscimol ([** ^**3**^ **H]‐9)**

^1^H NMR (300 MHz, MeOD‐D_4_) δ 7.48–7.30 (m, 5H), 6.00 (t, *J* = 0.9 Hz, 1H), 5.22 (s, 2H), 3.80 (d, *J* = 0.9 Hz, 0.3H), 3.74 (d, *J* = 0.8 Hz, 0.3H).

^3^H NMR (320 MHz, MeOD‐D_4_) δ 3.82 (d, *J* = 0.9 Hz, 0.28H), 3.76 (d, *J* = 0.9 Hz, 2H).

LRMS (ESI+): 205.1: 14.23%, 207.1: 64.13%, 209.1: 100%, 210.1: 12.82%.

**[** ^**3**^ **H]muscimol**·**HBr ([** ^**3**^ **H]‐3)**

^1^H NMR (300 MHz, D_2_O) δ 6.20 (s, 1H)

^3^H NMR (320 MHz, D_2_O) δ 4.17 (s, 0.23H), 4.13 (s, *J* = 0.8 Hz, 2H).

LRMS (ESI+): 115.1 (11.87%), 117.0 (28.64%), 119.0 (100%), 120.0 (7.22%).

Chromatography conditions:

METHOD 1:

Column: Luna 5 μm Phenyl‐Hexyl 100 Å, 250 × 10 mm.

Mobile phase: (A) 0.1% TFA in H_2_O, (B) 0.1%TFA in ACN, linear gradient from 90%A to 30%A over 40 min, followed by 5 min of washing with 10%A and 15 min of equilibration. Flow 4.7 mL/min.

METHOD 2:

Column: Luna 5 μm HILIC 200 Å, 250 × 10 mm.

Mobile phase: (A) 10 mM ammonium formate in H_2_O, (B) 10 mM ammonium formate in ACN/H_2_O 9/1, linear gradient from 2%A to 25%A over 30 min, followed by 5 min of washing with 25%A and 25 min of equilibration. Flow 1 mL/min.

## Conflicts of Interest

The authors declare no conflicts of interest.

## Data Availability

The data that support the findings of this study are available from the corresponding author upon reasonable request.
